# Resilience Among Undocumented Migrants: An Analysis of the Likelihood to Participate in a Panel Study Among Undocumented Migrants Amidst the COVID-19 Pandemic

**DOI:** 10.1177/00469580251324378

**Published:** 2025-03-17

**Authors:** Jan-Erik Refle, Yves Jackson, Claudine Burton-Jeangros

**Affiliations:** 1 LIVES, Swiss Centre of Expertise for in life course research, Geneva, Switzerland; 2 University of Geneva, Geneva, Switzerland; 3 Geneva University Hospital and University of Geneva, Geneva, Switzerland

**Keywords:** COVID-19, attrition, resilience, undocumented, vulnerability

## Abstract

Undocumented migrants, known for their particularly vulnerable living conditions, were deeply affected by the COVID-19 pandemic. Despite the challenges, some demonstrated resilience, successfully remaining in their host countries. Drawing on insights from the Parchemins study, which examined the experiences of undocumented and newly regularized migrants in Geneva, Switzerland, this paper details on undocumented migrants that faced accumulated difficulties during different moments of data collection amid the pandemic. Based on empirical findings, we observe that only a specific group among undocumented migrants continued to participate in the study post-pandemic. This paper undertakes a comparative analysis of “remainers” and those who dropped out. This indicator serves as a proxy for understanding resilience among the most vulnerable in times of crisis. Through regression models, we identify the factors influencing the likelihood of remaining in the panel as undocumented even in the face of a major crisis that negatively impacted various life domains. Our analysis reveals that the chance of remaining in Geneva amidst the pandemic was influenced by solidarity bonds, prior migration experience, as well as income developments and housing situations.

Prior migration experiences, positive economic development, the role of solidarity bonds, and the housing situation as important for withstanding hardship.

## Introduction

The Covid-19 pandemic affected different segments of the population to varying degrees.^1,2^ Some individuals with preexisting social vulnerabilities experienced profound changes in their lives, leading to additional stress and challenges to overcome.^3,4^ This was notably the case for undocumented migrants^5^—migrants without legal status—who face cumulated challenges in their daily life to achieve basic subsistence while often being ineligible to receive support from public authorities. The pandemic, like it is the case during economic crisis, sharply increased the need for resilience and adaptative strategies.^6-8^ At the same time, some problems like mental health, related ones became visible not only during the pandemic, but also after the end of restrictions and especially among migrants.^9^ Building on theories about vulnerability,^10,11^ this paper describes the characteristics of undocumented migrants who managed to keep participating to a 4-wave panel study during the pandemic, an element used as a proxy for measuring resilience. As participating in a panel requires the necessary time and availability, this indicator is used for assessing profiles of those still participating.

The article is structured as a case study, utilizing unique data from the Parchemins study, which followed undocumented and newly regularized migrants longitudinally. We followed this population over 4 waves of data collection and as part of this paper, we focus on those who remained undocumented throughout the study. Notably, the characteristics of undocumented participants in the last wave of data collection, towards the end of the pandemic in Switzerland in 2021/2022, differ from previous waves, indicating a selection effect where individuals with specific traits remained in the study. This suggests that only the most resilient undocumented individuals managed to stay in Geneva after the pandemic and to participate in a scientific study, while others may have returned to their home country or relocated. We hypothesize that the capacity to stay and participate is a consequence of developments that happened many years before its manifestation. The study’s aim is to identify factors influencing resilience. As part of this aim, we assess socio-demographic variables of those who stayed in the panel and those who dropped out are compared. Staying in the panel is used as a proxy for resilience, albeit not perfect, given that it demonstrates the capacity to participate in an unrewarded scientific study even when facing already harsh living conditions. We use exploratory factor analysis to first identify associations before testing the decisive factors for staying in the panel in a binary logistic regression model.

The paper starts with an overview on literature on vulnerability and undocumented migrants, before assessing the methodological aspects on attrition in panel research.

## Background and Literature on Undocumented Migrants and Vulnerability

Theories on the vulnerability of undocumented migrants generally focus on the specific conditions under which migrants live. Vulnerability describes a lack or loss of resources that affects the capacity to avoid or cope with stressors, or to recover from them.^10,11^ This approach is interpreted broadly, so that life events can positively or negatively affect the capacity to handle these stressors. A vulnerable population accumulates several factors that reduce its capacity to handle stressors, such as limited financial resources, poorer health, marginalization, or discrimination. Consequently, these vulnerable populations are more likely to be impacted by crises such as the Covid-19 pandemic.^3,4^ While migrants in general are often seen as vulnerable,^12^ undocumented migrants face particularly harsh living conditions. Nevertheless, some of them develop capacities to withstand such situations.

From a methodological perspective, vulnerable populations can be hard to reach and study.^13^ This is particularly the case for undocumented migrants that generally hide from authorities and that are difficult to research.^14^ Still, individuals who grew up in their country of origin, then moved to a second country, stayed there as undocumented for years, and finally encountered the pandemic, present a very specific profile for life course studies. Having developed characteristics that allow them to withstand numerous stressors simultaneously, such as frequent changes in housing situations, continuous hiding from authorities, or facing low salaries and unprotected working conditions, their study offers valuable insights into resilience amidst adversity.

### Migrants in a Life Course Perspective and Their Vulnerability

From a life course perspective,^15-17^ the lives of undocumented migrants are shaped by major life events. The act of migration represents a significant life event that alters many aspects of migrant workers’ lives. Furthermore, living as an undocumented individual includes a unique situation of vulnerability due to lack of work-related protections in terms of employment conditions and remuneration,^6^ unstable housing situations,^18,19^ the necessity to evade controls^20^ or the inability to visit relatives in the home country.^21^ These restrictions demand high flexibility from undocumented workers while also imposing a mental burden that can lead to high levels of depression.^22^

Given the vulnerability of undocumented migrants, the Covid-19 pandemic had significant effects on their lives.^23^ Many experienced reduced workloads and consequently lower household income levels.^4^ Their ability to withstand adverse events is further diminished, notably in terms of financial means, as undocumented migrants often lack the ability to accumulate savings.^6^ Ambrosetti et al furthermore underscored that the number of migrants among psychiatric emergency admissions was particularly higher after the governmental restrictions have been lifted.^9^ In line with findings on lower adherence to psychiatric treatment among non-Western migrants in Europe,^24^ it can be assumed that there will be long-term problems for a part of this population.

The literature on resilience among migrant populations offers insights into the factors that influence individuals’ ability to endure adverse life events. For instance, Cardoso and Thompson^25^ suggest that Latin American migrants often exhibit high levels of resilience, which can be attributed to various factors such as individual characteristics, family support, cultural aspects, and community assistance. Among these individual characteristics are competence in navigating difficult circumstances, alongside traits like self-esteem, self-mastery, and agency.^30^ Garcini et al^26^ have identified social support, spirituality, as well as cognitive and behavioral strategies as significant contributors to resilience. However, it’s important to note that not all individuals within migrant communities possess the capacity to withstand such pressures.

### Continued Participation or Dropout: Some Possible Reasons

Recruiting and maintaining undocumented migrants in a panel study is difficult due to their vulnerability and the characteristic as hard-to-reach population.^17^ Maintaining them in a panel during a pandemic becomes even more complicated, because individuals might react differently or even return to their country of origin. However, there is insufficient knowledge on coping mechanisms—especially in times of crisis—, as well as on reaction and eventually return strategies of undocumented migrants,^27^ our understanding of how undocumented migrants react when facing a major crisis such as the pandemic is limited. The challenge is identifying possible mitigation or exit strategies where scientific evidence is low: Scientific monitoring typically ceases once migrants leave a region or border, even if they were included in studies before departure. Thus, knowing details about the reasons for leaving a region or country or for not being able to participate any more are often not known.

The limited existent research basically showed 3 different mitigation or exit strategies: Relocating within the same country, returning into the country of origin or cutting expenses. Relocating to another region or country potentially alleviates economic pressure by leaving debts behind. Indeed, a study from the U.S. suggested that relocating within the country of work or returning to the country of origin might be viable options during times of crisis.^28^ But this research similarly showed that relocation must be carefully reflected decision and that another option is to basically cut all expenses that are seen as not essential during an economic crisis.^35^ Although this may be challenging as financial capacity is normally already low, it could result in losing stable housing or increased reliance on charitable assistance. Similarly, individuals may opt to work longer hours or take on riskier jobs, potentially hindering their participation in studies while remaining in the area.

Returning to the country of origin may still have been a strategy during the pandemic, as many countries offered repatriation flights at little or no cost.^29,30^ Indeed, some researchers report that the extent of return migration peaked during the pandemic.^34^ Research from the U.S. highlights migrants dropping out of the labor market for either a temporary period or permanently, with those in the bottom income tertile being more likely to emigrate eventually.^31^ However, Bhimji,^35^ when interviewing undocumented migrants in the U.S., also reported that returning without any money was not an option either.

### Attrition in Panel Research

Attrition in panel data research is a well-recognized phenomenon.^32-35^ It becomes problematic when it generates bias toward specific factors, particularly among vulnerable populations, leading to their underrepresentation in panel data. Overall, information on panel attrition during the pandemic is relatively scarce. Some examples suggest that young people are less likely to continue participation or that patterns are unsystematic.^36^ Research using the Dutch household panel (LISS) showed that especially the working population and women might be more susceptible to dropout.^37^ The finding on higher attrition among the workforce, particularly vulnerable to the pandemic, was also observed in a Canadian context.^38^ Rothbaum and Bee^39^ demonstrated in the U.S. context that non-response was higher among low-income populations during the pandemic. Additionally, in the U.S., Yu et al^40^ highlighted the role of age, with younger people dropping out, as well as ethnicity, among other factors. However, information on factors influencing dropout during the pandemic remains overall scarce and inconsistent, and no data on dropout among undocumented migrants could be identified in the existing literature. Thus, our theoretical assumptions about reasons for dropout and the factors influencing whether undocumented migrants manage to “survive” in harsh living conditions remain relatively thin, although literature about the general population informs us that incomes and employment might be influential factors for persisting participation in panel studies in times of crisis.

## Method

We followed STROBE^41^ combined guidelines for the study as the Parchemins study is a prospective study that followed individuals over several years.

### Selection and Description of Participants

Within this paper, we differentiate between individuals who continue to participate and those who are no longer engaged in the Parchemins study in line with attrition resulting from the pandemic. The Parchemins study accompanied undocumented and newly regularized migrants in line with a special regularization scheme in the Swiss Canton of Geneva. As part of this paper, we only focus on those that remain undocumented throughout the study. As evoked beforehand, the exact reasons for drop-out are unknown, but we take continuation as a proxy for individuals who found a strategy to withstand. In our case, this is furthermore context-dependent, as Switzerland experienced a period with many restrictions that consequently impacted the lives of undocumented migrants, followed by a later lifting of all restrictions. Specifically, the pandemic affected Switzerland between 2020 and 2021, and restrictions such as Covid certificates were definitively abandoned in 2022.^42^ In our case, the pandemic intervened during the third wave of data collection and data collection for wave 4 includes the time where restrictions in Switzerland where much lower. For our comparison, we focus on the dropout from the study between waves 3 and 4 and analyze this attrition in more detail.

The paper primarily relies on descriptive statistics due to the limited sample size within this highly specific population; yet endeavors to extend its analysis wherever feasible. The data derives from the Parchemins study, a panel study that tracked undocumented and newly regularized migrants across 4 waves of data collection spanning from 2017 to 2022. Initially, 468 migrant workers (undocumented and newly regularized) were enrolled, but due to attrition, this number decreased to 260 in wave 4 in 2022.^17^ While the comprehensive study configuration is detailed elsewhere,^6^ the focus of this article centers on undocumented migrants who remain undocumented over time (ie, the study control group) and does not include those that become regularized. This includes overall 203 migrants were our last information before leaving or during the last wave is that they did not have a residence permit.

### Data Collection and Measurement

The data collection was conducted simultaneously with a cantonal regularization scheme, targeting undocumented migrants who had been residing in Geneva for several years. The study protocol was approved by the Ethics Committee of the Canton of Geneva, Switzerland (authorization). To ensure a population of undocumented migrants comparable to those who were finally regularized, a participation criterion was set for the study: individuals had to have lived in Geneva for at least 3 years and expressed a desire to stay for an extended period. Among the undocumented participants in the study were some who were ineligible for regularization because they did not meet the minimum 10-year residency requirement without children at the time of the scheme in Geneva (2017-2018). Others, who were originally undocumented, later obtained a residence permit through the scheme. The Parchemins data exclusively focuses on undocumented and newly regularized migrants, both of whom still exhibit various elements of vulnerability.^6^

Acknowledging that undocumented “survivors of the pandemic” present a different profile compared to those that are no longer available for a survey, it is worthwhile to shift perspective toward understanding the profiles of these individuals and the factors that set them apart. The Parchemins study invested significant resources to retain as many participants in the panel as possible, even during the challenges posed by the pandemic,^17^ notably to avoid specific profiles getting lost over time. As outlined in the theory section, the focus is on the most resilient individuals among a particularly vulnerable group: Undocumented migrants who persist in staying in Geneva throughout the study period and remain engaged in the panel despite the challenges posed by living undocumented and enduring the pandemic. We assume that the pandemic acted as a selective event that influenced our population under study. In the Parchemins study we do possess baseline socio-demographic profiles before they interrupted their participation. While this does not shed light on exit strategies or subjective motivations for dropout, it does provide insights into who remains in the host country *and* possesses sufficient time and resources to participate in scientific research projects.

Summarizing reasons for dropout is difficult and the present study is no exception regarding information on why participants left the panel. As we lack information on the circumstances surrounding undocumented migrants’ decision to discontinue their participation, we refer to the identified differences in terms of profile when comparing undocumented migrants still present in wave 4 and those observed in earlier waves. These individuals in wave 4 are in a comparatively better socioeconomic position.^6^ This observation serves as the foundation for the article, raising questions about the characteristics and circumstances of these “remainers” in wave 4.

We are interested in the differences between undocumented migrants who definitively left the panel and those who remained during and after the pandemic. To achieve this, we created a dichotomous variable: coded 0 for those who left the panel across the first 3 waves of data collection (n = 131) and coded 1 for those who remained in the panel in wave 4 (n = 72). Facing the additional challenge of lacking theoretical expectations about the “remaining” of undocumented migrants, we opted for a rather unorthodox and inductive approach. We relied on exploratory factor analysis to determine which variables to include and eventually test in a regression model. We document this process in detail, as there is limited knowledge on the factors influencing panel attrition among undocumented migrants—especially in times of crisis—, making the documentation of relevant variables crucial for future studies.

The factor analysis focused on the dependent variable to identify factors explaining remaining in the panel over several years and under harsh living conditions. Moreover, the very low sample size, often under 200 respondents due to missing data for some variables, added to the challenge. The factor analysis, conducted pairwise, and correlation table identified variables with a correlation value of at least .100. Several rotations were checked, resulting in a reduction of the number of relevant variables that were originally available in our dataset as outlined below. The results of this procedure are presented below and build the basis for the regression model.

Regarding the binary logistic regression, we use selected variables from the exploratory factor analysis as dependent variable and introduce these in a regression model. As part of this model, supplement with socio-demographic information. One of the selection criteria was the aim of reducing the number of missing values. In case of several variables with quasi-equal correlation values, a preference was given to those with more responses.

We however underscore that we refer to information from wave 1 in order to provide information for all that leave over the data collection period. Obtaining more detailed information on who stays after several years of data collection and during an intervening pandemic among undocumented migrants is challenging without further reducing the sample size. All analyses were conducted using SPSS.

### Statistics

We are interested in a comparison of migrants that remain undocumented throughout the study period and those who dropped out between waves 1&2, 2&3, or 3&4. We expect that there is an additional economic pressure due to the pandemic that starts in wave 3 and is still observable in an accentuated form in wave 4. Table 1 gives an overview on the populations studied by this paper. The initial population of undocumented migrants—however—gets in part regularized over the course of the study and holds different characteristics compared to those that stay undocumented. We still present it for general comparison. The information on dropouts stems from those with definitive drop-out only and does not account for those that returned to the study over time. The number of returners—notwithstanding whether undocumented or regularized—does however only include 11 persons in the study.

**Table 1.** Distributions for Key Variables Among Undocumented at the Beginning and at the End, as well as Definite Dropouts by Wave.

	Undocumented W1 that later dropped out or still participated in W4	Dropout W2	Dropout W3	Dropout W4	Remainers W4
N	203	70	36	25	72
Age (Average) (years)	42	42	44	46	44
Sex (%Women)	67	64	64	64	72
Duration of stay at baseline (average, years)	10	10	11	10	9
Having lived in another country than the country of origin before coming to Switzerland (%)	19	13	14	20	27
Origin (%)					
Eastern Europe	12	17	11	20	6
Latin America	58	51	64	56	63
Africa	12	6	14	4	11
Asia	17	21	11	20	21
Education level (%)					
Up to primary school compl.	10	16	11	4	6
Up to secondary school compl.	68	60	72	80	70
Higher education	22	24	17	16	24
Approximation of available household income in CHF (Median)	1900	1800	2000	1600	2000
Type of housing contract (%)					
Sublet	79	76	71	87	63
Accommodation at employer	7	6	0	0	14
Rental contract	14	18	29	13	23
Sector of activity (%)					
Domestic work	70	69	72	63	70
Construction	7	9	9	16	3
Hospitality	11	11	6	5	8
Other	12	11	13	16	19
Renounced healthcare due to costs (%)	30	19	42	39	32
Support from relatives (only measured in W1 and W4) (%)	77	71	72	72	81

*Note.* % rounded. For those who dropped out, the last available information is shown, which refers to the previous wave. For the initial undocumented individuals as well as those who remained, the then available information is presented. People who paused their participation are not included, only definite dropouts. Among the undocumented are also those who have applied for a permit but for whom no decision has been made.

As shown in Table 1 and on average, the individuals who remained in the study were younger at the beginning of the study and had been living for a shorter time in Geneva. Those remainers who did not submit any application for regularization over time were all living in sublets, while those who applied but had not yet received a response were more often living in apartments with their own rental contracts. Notably, individuals with higher levels of education were less likely to drop out during the pandemic (waves 3 & 4). Additionally, support from relatives was higher among those who remained in the study compared to those who dropped out over time.

Despite these observations, many tendencies remain inconclusive. Dropout appears to be associated with multiple factors, with changes becoming particularly evident with the onset of the pandemic, especially in wave 3. Particularly noteworthy is the influence of renouncing healthcare due to costs during the pandemic. Those who dropped out in waves 3 and 4 exhibited higher levels of healthcare foregoing compared to undocumented individuals at the beginning of the study as well as compared to remainers. To approach more robust results, we then tested the variables available in our dataset in line with an exploratory factor analysis.

## Results

### Exploratory Factor Analysis

As part of the above-described procedure, we first identified correlates that have a value higher than 0.100 in order to identify relevant variables for a regression model. The exploratory factor analysis identified several key variables correlated with remaining in the panel. These include having health insurance, receiving health insurance subsidies, having a family doctor, renouncing healthcare, and the number of hospitalizations. Self-reported depression is another correlated variable. Additionally, having lived in a country other than the country of origin before coming to Geneva and the length of stay in Geneva are relevant. Practical support from relatives, marital status, body mass index, and accommodation costs also play a role. Unsuccessful job search attempts, household equivalent income, individual salary, and cumulative household salaries are further variables identified. Selected mental health indicators include GAD-7, item 5, which asks about being so active that it’s difficult to calm down, the Maslach Burnout Inventory item on feeling emotionally exhausted by, and PHQ-9, item 3, which asks about having difficulty falling asleep or staying asleep, or sleeping too much. Participants who remained in the study were characterized by lower healthcare consumption, exhibit more self-reported depression, stayed for shorter durations, were more likely to having lived in another country before, had more support during the first wave of data collection, were more likely to be divorced, exposed a higher body mass index, had a lower rent, and experienced more unsuccessful job searches in wave 1. Those with lower income in wave 1 also have a higher chance of staying, as do those who report difficulties in falling asleep and feeling emotionally exhausted. Regarding income, those who remained in the panel reported, on average, an increase of their equivalent household income of 506 CHF compared to wave 1, while those that left either before waves 4 or 3 reported in average losses of 200 CHF compared to wave 1 before dropping out. These findings point to economic dynamics that manifest up to several years later.

We also ran additional models to check whether the continent of origin, sex, sector of activity, or educational level played a role, but this was never the case. In fact, we ran many different analyses with inconclusive findings, mostly due to missing significance.

### Binary Logistic Regression Model

As a final step, we tested the variables in the binary logistic regression model, contrasting those who remained in the panel (coded 1) until wave 4 with those who dropped out during the data collection (coded 0). Only questions with the most answers were included in the model, to maximize the number of responses. Additionally, we utilized information from Wave 1, which is several years old by the time of Wave 4. Table 2 provides an overview of the findings. The model should be interpreted with caution due to the low sample size.

**Table 2.** Binary Logistic Regression on Factors Associated With Staying in the Panel in Wave 4 (1) Versus Early Drop-Out (0).

	Coeff. (Std.-Err)
Lived in another country than the country of origin before coming to Switzerland	0.90 (0.46)*
Approximation of the equivalent monthly disposable household income	0.00 (0.00)
Renounced to healthcare due to costs, last 12 months no/yes	0.833 (0.39)*
Self-reported chronic diseases: Depression or anxiety no/yes	0.71 (0.45)
Practical support from relatives	1.39 (0.47)**
Accommodation: Total monthly rent, including service charges paid by the household	−0.00 (0.00)**
Body mass index—score	0.06 (0.04)
Partnership status (no/yes)	
Single^a^	−0.67 (0.49)
Married^a^	−0.78 (0.60)
Unsuccessful recent attempts to find a new job (no/yes)	0.58 (0.38)
GAD-7: In the past 2 weeks, how often have you been disturbed by the following: Active so that it’s difficult to calm down	−0.02 (0.23)
Constant	−2.87 (1.25)*
*R* ^2^	.25
n	180

a Reference category separated/divorced/widowed, the information shown derives from the first wave of data collection in order to maximize the number of respondents included.

*** *P* < .001. **P < .01. *P < .05.

The explained variance of the model is high, accounting for approximately 25%. When examining the factors, it becomes evident that receiving practical support from relatives played a crucial role in remaining in the panel. Another significant factor was whether the undocumented migrant had lived in a country other than their country of origin since leaving it; those with prior migration experience were more likely to stay. Renouncing healthcare in wave 1 also had a significant impact on individuals’ decision to continue participating. The effect of monthly rent paid on remaining in the study tends toward zero, with those paying lower rent more likely to stay until the end of the study. Interestingly, household income was not found to be significant in this model. As this information stems from wave 1 it does however not account for the increases or decreases in income since as described above.

As part of robustness checks, we conducted several additional models where prior migration experience was sometimes found to be not significant or only marginally significant. Our regression model here comes to its limits. We adopted this approach using data from an earlier wave to increase the sample size, but it does not consider developments that occurred since the beginning of the study, including the pandemic. Despite several attempts to identify additional factors, beyond those presented above and such as comparing those who left and stayed across different waves, we did not find any further meaningful results.

## Discussion

Receiving support from family and relatives influences the ability to sustain participation in a longitudinal study. This factor consistently emerged across our analyses and appears to bolster resilience against adverse living conditions. The importance of such support mechanisms during the pandemic has also been demonstrated in other research,^43^ although not specifically in the context of undocumented migrants. Furthermore, it suggests that these individuals received support that included material or economic resources, as undocumented migrants without a job do not have access to welfare support. Although this restriction was alleviated during the COVID-19 pandemic, many still emphasized their preference for finding private solutions rather than seeking institutional support. This also suggests that this specific group is not isolated.

Presenting this finding more cautiously, we also observe that previous migration experience increases the likelihood of remaining during times of crisis. This suggests that prior migration experience may provide individuals with coping mechanisms, enabling them to better withstand crises that exacerbate adverse living conditions. It is noteworthy that while prior migration experience appears to have negative effects on, for example, mental health when compared with first-time migrants,^44^ the specific experience of prior migration could be beneficial in times of crisis. On the one hand, it could suggest that they are more enduring undocumented migrants: having the will to find better opportunities (selection process) or on the other hand that returning is not an option for them since they preferred to move to another country than return home in the past and have not worked in the country of origin since a longer time. Still, we do not know, how remaining in Geneva evaluates compared to strategies such as leaving or relocating.^35^

Central to, and aligned with the marginal finding of prior migration experience is the question of mitigation or exit strategies applicable during crises, which relates to successive stages of migration. Generally, research has identified the perspective of multiple migration as gradually improving living standards, as well as the perspective of migration as a chain of precarity where precarity continues in another location.^45,46^ While our data does not provide a definitive answer, it could suggest that multiple migration leans more toward chains of precarity and only yields positive effects during additional crises. However, given the multitude of events that influence the lives of undocumented migrants, it remains unclear whether central factors supporting or opposing this theory can be extracted, even with better data. This however, does not change that undocumented migrants suffer mentally from long-term consequences of precarity.^26,47^

Of note, our final regression model solely relies on information from wave 1. Consequently, we are uncertain whether the support from relatives only applies to the initial phase during wave 1 of data collection or if there is ongoing support, or selective support in case of problems, from relatives. As research emphasizes support networks,^26,48-50^ it would be interesting to explore whether some form of “reverse” remittances contributed to a more stable position for the remaining undocumented individuals. For instance, Mazzucato^51,52^ described “reverse” remittances typically as services by those that receive the remittances in the country of origin and that thus avoid sending more money to the country of origin. A similar argument is made by Yeboah et al.^53^ Another form of “reverse remittances” might involve debts taken in the country of origin and transferred to the country of work.^54^ Ran and Liu,^55^ however, also described the possibility of older family members sending money to their relatives in the working country. Existing literature mainly refers to services provided in the country of origin and rarely discusses whether, in difficult times, the re-transferal of saved money sent to the country of origin, such as for retirement, to the working country is an option. As savings in the country of work are generally absent among undocumented migrants, tapping into reserves—or existing banking services in another country—could be a viable solution to bridge short moments of uncertainty. However, at the beginning of the pandemic, it was unclear how long this event would last.

Although the sample size is small and the indicator weak, regression models show the critical role of housing. During the COVID-19 pandemic, housing emerged as the most significant fixed cost for undocumented migrants. Subletting or sharing flats with more people provided a means to alleviate this burden, enabling participants to maintain a certain level of financial stability despite reduced income. Still, subletting comes with the adverse effect that consequences for wellbeing and perceived autonomy might be negative. While our knowledge in this area is incomplete, insights from regularized migrants highlight the importance of housing autonomy post-regularization. Contrasting those who dropped out earlier, it is evident that remaining undocumented migrants face housing instability, with very few having formal lease agreements.

Another noteworthy observation concerns variables correlating with dropout but being not significant in regression models. While mental health indexes showed no direct influence, specific subcomponents such as emotional exhaustion and sleeping difficulties correlated with remaining in the panel. This suggests an emotional attachment to work, possibly explaining why some undocumented migrant workers who remain in the panel reside in their employers’ homes. The limited literature suggests that emotional attachment influences length of stay and ties into the emotional aspect of care work.^56^

All these findings, while intriguing, are based on a small and specific sample and warrant validation in other contexts. For example, healthcare consumption’s potential role in sustaining panel participation and undocumented status raises questions about the unforeseen costs it may entail, particularly during a pandemic.

There are a number of limitations to our study. With respect to our data, a larger sample and better opportunities for analyzing data on undocumented migrants are desirable, including for longitudinal models such as fixed effects models. However, as it is a hard-to-reach population, it is doubtful whether panels of similar size are realizable. Another factor that would be interesting to observe—especially for analysis crises reactions—are personality traits (eg, Big 5 or other models,^57^ of those that remain over the years. However, at least in our data, we do not have this information. Finally, our proxy of staying in the panel as equal to resilience is not perfect. The main weakness remains our incapacity to know more about undocumented migrants that drop out. Their dropout can have many different reasons, but our analysis tends to show that attrition in times of crisis is not purely random. Notwithstanding, we do not know whether undocumented migrants that stay in an area are finally better off compared to those who relocate, be it economically or in terms of health or housing.

## Conclusion

By using unique data from a panel study, we compare undocumented migrants that still respond after 4 waves of data collection (2 before, 2 after the pandemic) with those that drop-out earlier. Our findings show the importance of support from family and relatives, prior migration experience as well as the housing situation. The latter is in line with overall income levels. Additional analysis shows the relevance of healthcare consumption and of some cognitive mechanisms in terms of emotional attachment that deserve further research efforts. At the same time, our study shows the ongoing need to monitor the population of undocumented migrants and especially to design studies that can follow those are currently not able to participate in a scientific study following years of prior participation. In fact, we know probably best about undocumented migrants with comparatively better living conditions, and much less about those that live under the most severe conditions. The findings also recall that major crises such as the pandemic impact the most vulnerable of all.
